# The association of immunosurveillance and distant metastases in colorectal cancer

**DOI:** 10.1007/s00432-021-03753-w

**Published:** 2021-09-02

**Authors:** Sven Jacob, Vindi Jurinovic, Christopher Lampert, Elise Pretzsch, Jörg Kumbrink, Jens Neumann, Ren Haoyu, Bernhard W. Renz, Thomas Kirchner, Markus O. Guba, Jens Werner, Martin K. Angele, Florian Bösch

**Affiliations:** 1 Department of General, Visceral and Transplantation Surgery, University Hospital, LMU Munich, Marchioninistr. 15, 81377 Munich, Germany; 2 The Institute for Medical Information Processing, Biometry, and Epidemiology, Ludwig-Maximilians-University (LMU) Munich, Munich, Germany; 3 Institute of Pathology, Ludwig-Maximilians-University (LMU) Munich, Munich, Germany

**Keywords:** Colorectal cancer, Liver metastases, Peritoneal carcinomatosis, Hematogenous metastases, Gene signature

## Abstract

**Background:**

Colorectal cancer (CRC) is the third most common malignancy worldwide, but the key driver to distant metastases is still unknown. This study aimed to elucidate the link between immunosurveillance and organotropism of metastases in CRC by evaluating different gene signatures and pathways.

**Material and methods:**

CRC patients undergoing surgery at the Department of General, Visceral and Transplantation Surgery at the Ludwig-Maximilian University Hospital Munich (Munich, Germany) were screened and categorized into M0 (no distant metastases), HEP (liver metastases) and PER (peritoneal carcinomatosis) after a 5-year follow-up. Six patients of each group were randomly selected to conduct a NanoString analysis, which includes 770 genes. Subsequently, all genes were further analyzed by gene set enrichment analysis (GSEA) based on seven main cancer-associated databases.

**Results:**

Comparing HEP vs. M0, the gene set associated with the Toll-like receptor (TLR) cascade defined by the Reactome database was significantly overrepresented in HEP. HSP90B1, MAPKAPK3, PPP2CB, PPP2R1A were identified as the core enrichment genes. The immunologic signature pathway GSE6875_TCONV_VS_FOXP3_KO_TREG_DN with FOXP3 as downstream target was significantly overexpressed in M0. RB1, TMEM 100, CFP, ZKSCAN5, DDX50 were the core enrichment genes. Comparing PER vs. M0 no significantly differentially expressed gene signatures were identified.

**Conclusion:**

Chronic inflammation might enhance local tumor growth. This is the first study identifying immune related gene sets differentially expressed between patients with either liver or peritoneal metastases. The present findings suggest that the formation of liver metastases might be associated with TLR-associated pathways. In M0, a high expression of FOXP3 + tumor infiltrating lymphocytes (TILs) seemed to prevent at least in part metastases. Thus, these correlative findings lay the cornerstone to further studies elucidating the underlying mechanisms of organotropism of metastases.

## Introduction

Colorectal cancer (CRC) is the third most common malignancy worldwide with an increasing incidence over the past decades. Annually approximately 1 million new cases are detected (Sakin et al. 2019; Arnold et al. 2017). Moreover, all over the world more than 15% of all cancer-related deaths are attributed to CRC (Siegel et al. 2019) with metastatic spread as the most significant prognostic factor (Cao et al. 2015; Ferlay et al. 2010). In this respect, up to 30% of CRC patients present with distant metastases at initial diagnosis. Furthermore, patients without distant metastases might have progressive disease along with metachronous metastases to the liver or the peritoneum in 60% of cases (Kim et al. 2015; Tauriello et al. 2017; Siegel et al. 2012). However, no specific markers or distinct gene signatures have been identified so far defining the risk for developing distant metastases.

Recently it has been demonstrated that various immunoreactive processes, including local inflammation, immunological response, changes in microRNA profiles, and differentially expressed oncogenes influence the formation of distant metastases (Pretzsch et al. 2019; Brenner et al. 2014; Strubberg and Madison 2017; Muhammad et al. 2014). Up until now however, the effect of immunosurveillance on the underlying mechanisms of metastasis formation and organotropism remain ill-defined. Recent evidence suggests that the adaptive as well as the innate immune system might be involved in the metastatic process. In this respect, tumor infiltrating lymphocytes (TILs) seem to influence tumor cell intravasation and other early metastatic events, such as epithelial mesenchymal transition (EMT) and intratumoral intravasation (Deryugina et al. 2020; Zhang et al. 2020). Thus, a deeper knowledge of immunology-associated oncogenes and their role in metastasis formation is required. Understanding the mechanisms of metastasis could help to develop personalized diagnostic and therapeutic approaches in the treatment of CRC patients.

Thus, the present study aimed to investigate gene signatures of locally advanced CRC leading to distant metastases. Furthermore, this study evaluated gene pathways and highlighted a potential crosstalk between genes involved in the process of metastasis via a large-scale gene set enrichment analysis (GSEA).

## Material and methods

Colorectal cancer patients surgically treated at the Department of General, Visceral and Transplantation Surgery at the Ludwig-Maximilian University Hospital Munich (Munich, Germany) were screened. Patients missing formalin-fixed paraffin-embedded (FFPE) tissue of the primary tumor, as well as patients presenting with co-malignancies, Lynch-Syndrom and other hereditary diseases were excluded from the present analysis. After a 5-year follow-up, the patients were grouped into three groups: patients with locally advanced CRC without metastases (M0), patients with distant metastases exclusively to the liver, either synchronous or metachronous (HEP) and patients with peritoneal carcinomatosis (PER). Follow-up was conducted by periodic visits and cross-sectional imaging was done after defined periods. To prevent possible overlaps, patients with hepatic and peritoneal metastases were not included in the present analysis. Locally advanced CRC was defined as T3 or T4 tumors, thus patients with a T1 or T2 tumor were not included in the present analysis.

Eighteen patients, six from each group, were randomly selected for further characterization. Primary tumor RNA was isolated from FFPE specimens via microdissection as described previously (Bösch et al. 2019a). NanoString analysis using the nCounter® PanCancer Progression Panel by NanoString-Technologies (Hamburg, Germany) was used (Tsang et al. 2017). Recently, it has been demonstrated that this panel generates reliable and valuable results in cancer research (Cheng 2020; Sundar 2020; Bilusic et al. 2021; Bugide et al. 2020). The panel includes genes (*n* = 770), which are grouped into main biological aspects such as angiogenesis, tumor growth and invasion, metabolism and hypoxia as well as epithelial-mesenchymal transition (EMT). Furthermore, layers of the extracellular matrix and its remodeling and distinct transcription factors were included in the present analysis. But genes not included in the nCounter^®^PanCancer Progression Panel are not detected by the present analysis. All genes were tabulated according to their level of significance and their gene expression value. Gene expression levels of M0 were compared to HEP and PER. The entire gene expression analysis was included in the primary GSEA which was performed using the Broad Institute software (Yang 2021). Results were referenced to pathways from the following databases: Reactome, Biocarta, Kyoto Encyclopedia of Genes and Genomes (KEGG), Gene Ontology (GO), Hallmark gene sets, oncogenic and immunologic signatures (Fabregat et al. 2018; Kanehisa et al. 2017; Resource 2019; Liberzon et al. 2015; Lin et al. 2007). The *p* values are considered nominal *p* values and were adjusted for multiple testing within the specific pathway data base with the Bonferoni-Holm method (*q* value). *p* values ≤ 0.05 and *q* values ≤ 0.25 were considered significant, *p* values < 0.001 were considered highly significant.

## Results

### Baseline patient characteristics

Six patients were included in each group. Medium age was 70.2 years (± 8.5 years). Eleven female and seven male patients were analyzed. The primary tumor site was equally distributed among the groups. The vast majority (> 85%) of carcinomas were staged as T3 with lymph node metastases. Of the 12 patients with metastases, 9 had synchronous metastatic disease and 3 patients developed metachronous spread. All patients analyzed received either neoadjuvant or adjuvant chemotherapy. A detailed overview of the entire cohort is given in Table 1.

**Table 1:** Baselines patient characteristics of the analyzed patients

Patient	Group	Gender	Age	PTS	T	N	M	G	UICC	M (s/m)	Therapy
1	PER	f	73	2	4	1	1	3	4	n.a	2
2	PER	f	72	5	4	1	1	3	4	n.a	2
3	PER	m	84	1	3	1	1	3	4	n.a	2
4	PER	f	48	1	3	0	1	2	3	n.a	2
5	PER	m	89	4	3	1	1	2	4	n.a	2
6	PER	f	61	2	4	1	1	2	3	n.a	2
7	HEP	m	70	4	3	0	1	2	4	s	3
8	HEP	m	76	6	3	2	1	3	4	s	2
9	HEP	f	78	2	3	1	1	2	3	m	2
10	HEP	f	74	1	3	2	1	3	4	s	2
11	HEP	m	56	6	3	1	1	2	4	s	3
12	HEP	m	57	6	3	2	1	3	4	s	2
13	M0	f	73	5	3	1	0	3	3	n.a	2
14	M0	f	67	3	3	2	0	3	3	n.a	2
15	M0	f	86	6	3	1	0	3	3	n.a	2
16	M0	f	59	2	3	1	0	2	3	n.a	1
17	M0	f	74	1	3	2	0	2	3	n.a	2
18	M0	m	67	4	3	1	0	2	3	n.a	2

Gender: *f* female, *m* male; *PTS* primary tumor site: 1 = coecum, 2 = ascending, 3 = transvers, 4 = descending, 5 = sigmoid, 6 = rectum; M (s/m): m = metachronous metastasis, s = synchronous; Therapy: 1 = neoadjuvant, 2 = adjuvant, 3 = both; G = grading; T = tumor size; N = lymph node status; M = distant metastasis; *UICC* union for international cancer control

### NanoString analysis

The NanoString-Analysis was performed using the nCounter^®^ PanCancer Progression Panel by NanoString-Technologies (Hamburg, Germany). This panel includes 770 genes associated with the development and progression of distant metastases. These 770 genes were cross-referenced with every pathway of the 7 above mentioned databases. Accordingly, pathways which include genes analyzed with the nCounter^®^ PanCancer Progression Panel were further evaluated by GSEA. The total number of potential pathways of each database as well as the number of tested pathways are displayed in Table 2.

**Table 2:** The potential pathways of the seven databases and the final pathways applied to large-scale gene set enrichment analysis

Database	Potential pathways (n)	Tested pathways (n)
Reactome	674	58
Biocarta	217	14
KEGG	186	57
GO	5917	1182
Hallmarks gene sets	50	24
Oncogenic signatures	189	76
Immunologic signatures	4872	1177

*KEGG* Kyoto encyclopedia of genes and genomes; *GO* Gene ontology

### Gene set enrichment analysis of HEP vs. M0

#### Reactome database

In tumors leading to liver metastases (HEP), the Reactome Toll Receptor cascade (RTRc) of the Reactome database was highly enriched. The normalized enrichment score (NES) of 1.68 had a significant q value of 0.23. RTRc was one of the 58 tested pathways out of the Reactome database and includes 118 genes. Out of these 118 genes 17 genes were included in the chosen NanoString-panel.

**Fig. 1:**
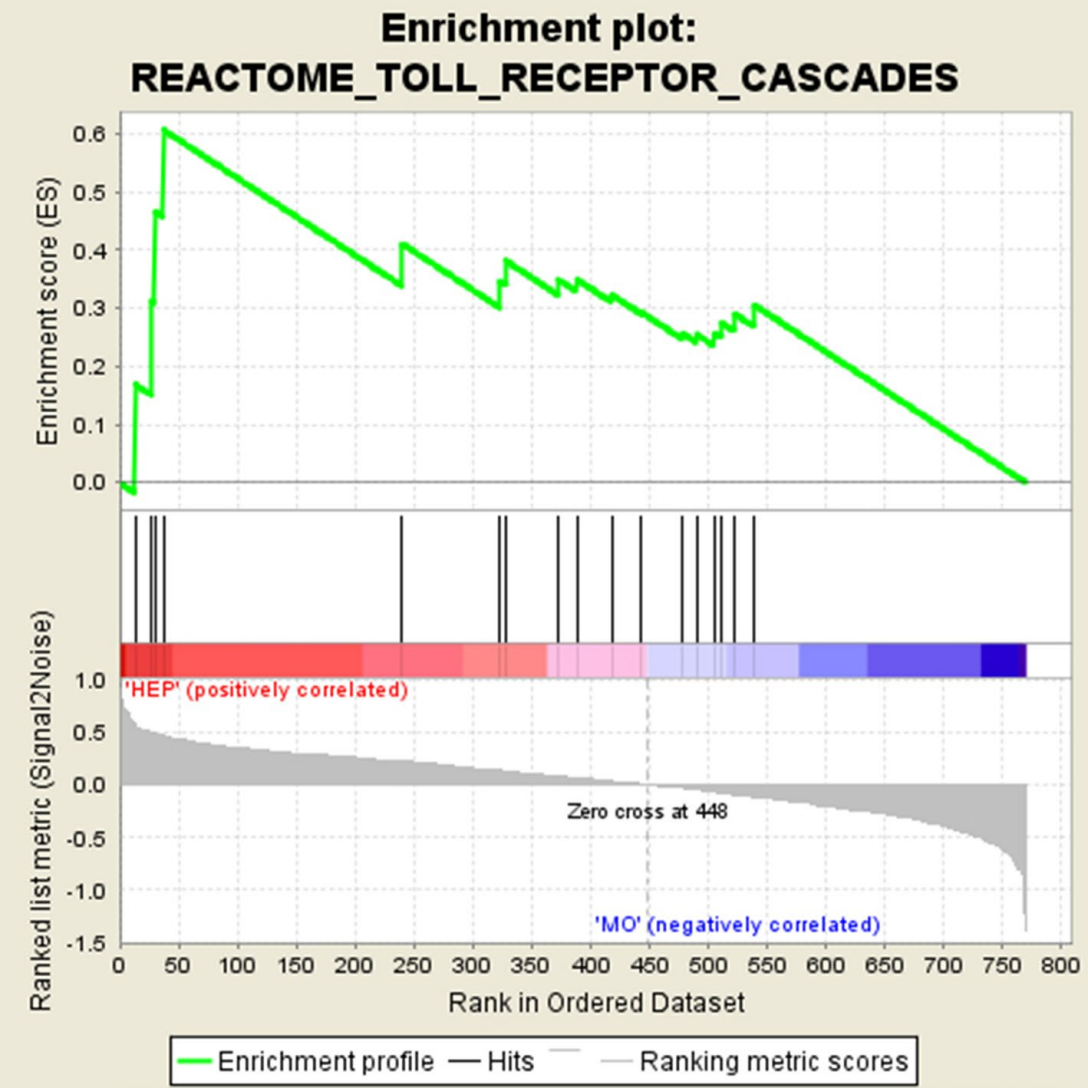
Enrichment plot for the Reactome_Toll_Receptor_Cascade from the Reactome database displaying the enrichment curve, graphical approximation of tested genes and their alterations from the base line (*p* value and *q* value)

**Fig. 2:**
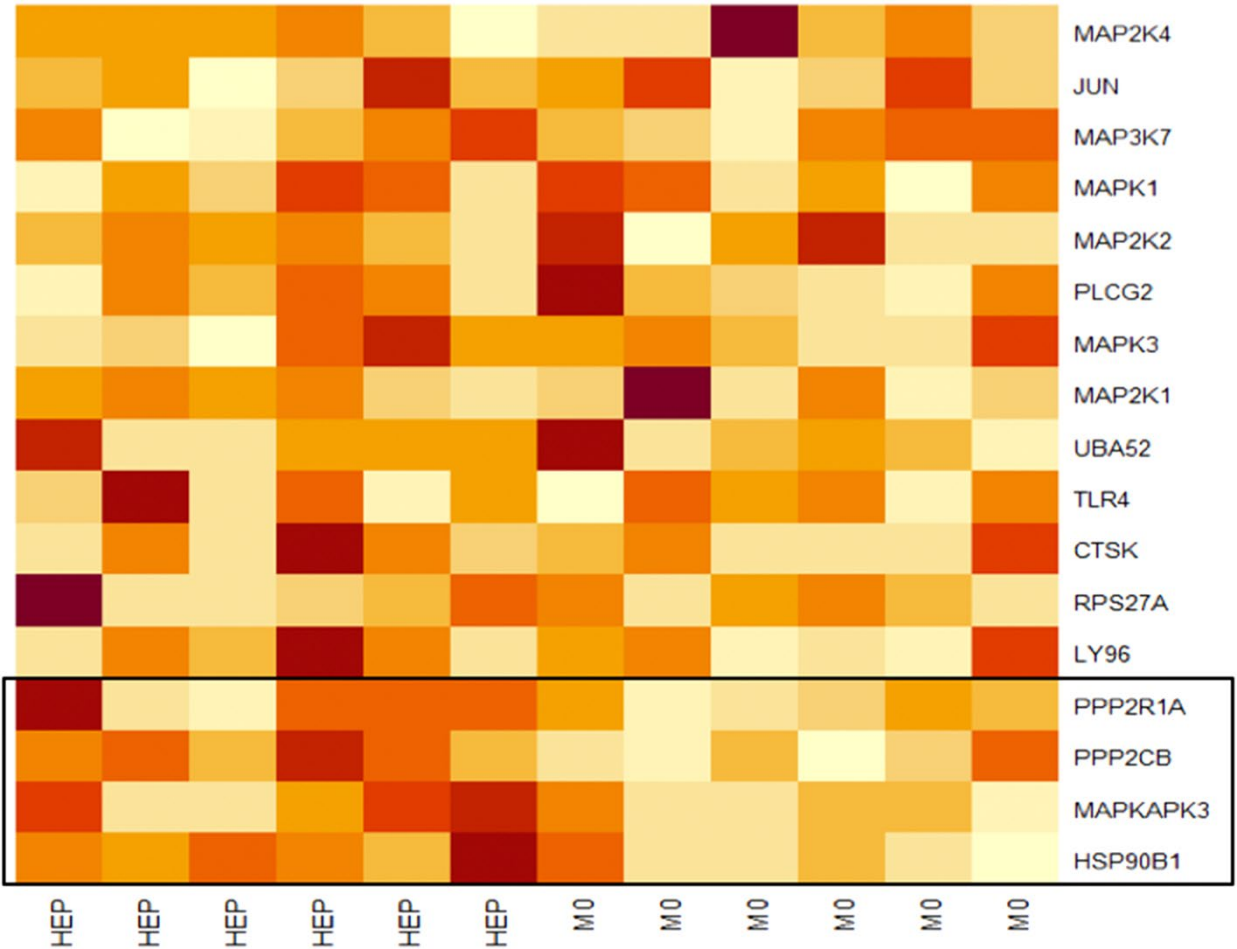
Heat map displaying all tested genes and their expression profiles from the Reactome_Toll_Receptor_Cascade of the Reactome database. Strong expression = dark red, semi-strong expression = orange, weak expression = white. Core enrichment genes are highlighted in the box

Consequently, these 17 genes were transferred to the enrichment analysis. Four specific genes namely HSP90B1, MAPKAPK3, PPP2CB, PPP2R1A exhibited the highest contribution to the NES of 1.68 indicating an important role in the gene set associated to Toll-like receptor (TLR) pathways and the development of liver metastases. These 4 as well as the other 13 tested genes are represented in the enrichment plot in Fig. 1. The contribution of genes to the enrichment curve is shown. All tested genes and their differential expression are presented in the heat map of Fig. 2.

#### Immunologic signature database

In locally advanced tumors without distant metastases (M0), the immunologic signature pathway GSE6875_TCONV_VS_FOXP3_KO_TREG_DN was remarkably overrepresented compared to patients presenting with liver metastases (HEP). The NES of −1.89 was highly significant (*p* < 0.001) with a significant *q* value of 0.16. From 200 genes in this pathway, 18 were also present in the NanoString-panel.

**Fig. 3:**
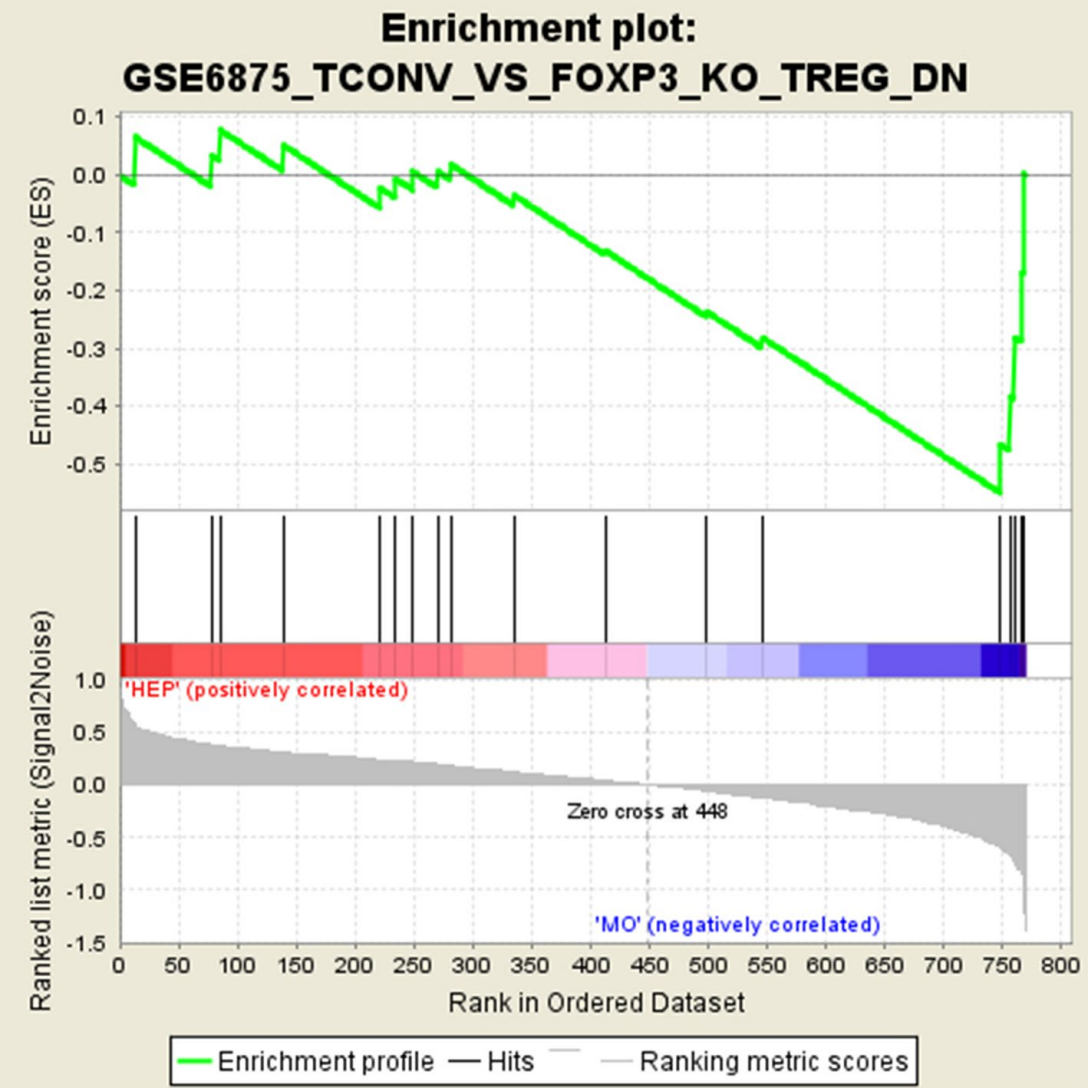
Enrichment plot for the GSE6875_TCONV_VS_FOXP3_KO_TREG_DN pathway the Immunologic signature database displaying the enrichment curve, graphical approximation of tested genes and their alterations from the base line (*p* value and *q* value)

**Fig. 4:**
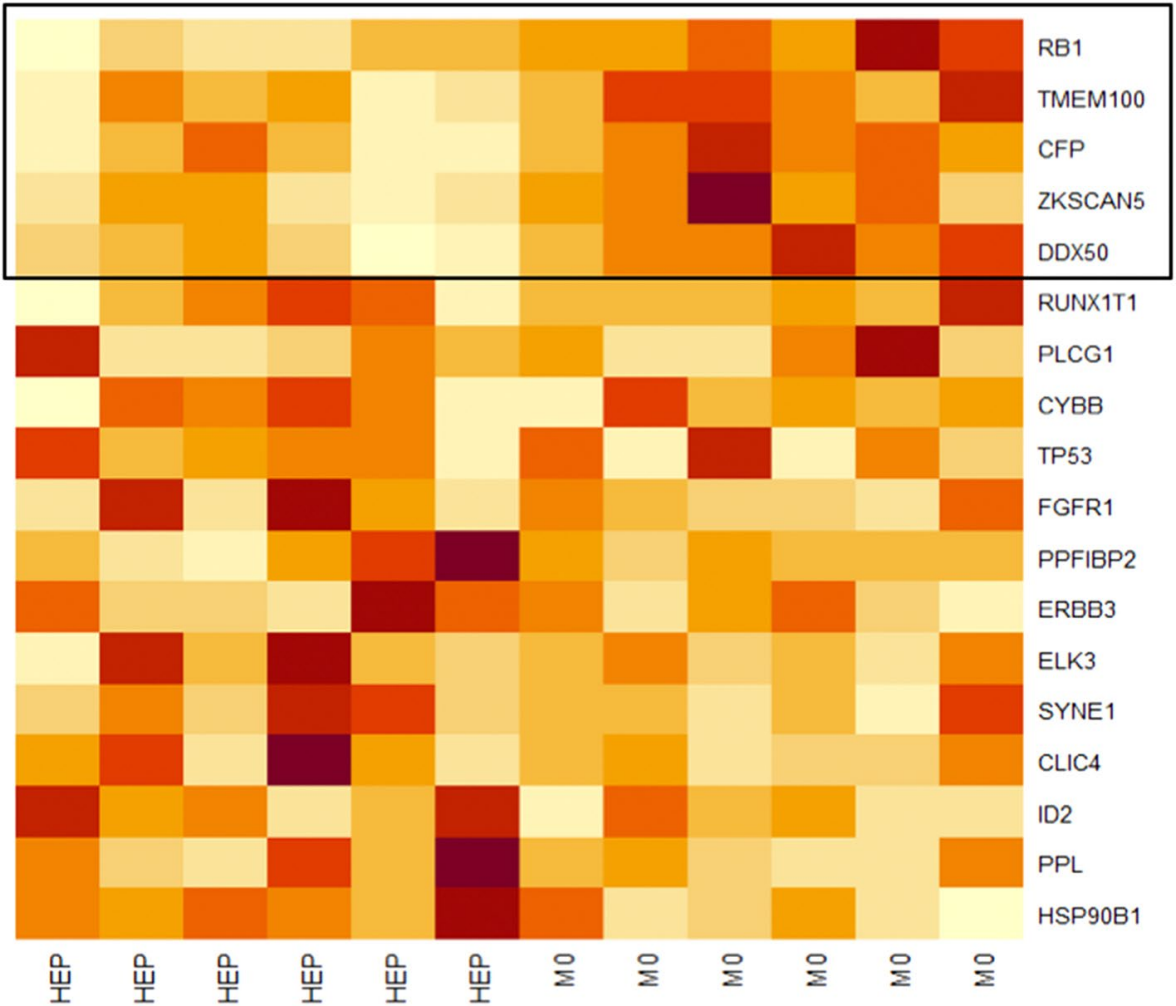
Heat map displaying all tested genes and their expression profile from the GSE6875_TCONV_VS_FOXP3_KO_TREG_DN pathway of the Immunologic signatures database. Strong expression = dark red, semi-strong expression = orange, weak expression = white. Core enrichment genes are highlighted in the box

From those 18 tested genes, 5 genes (RB1, TMEM100, CFP, ZKSCAN5, DDX50) had the greatest influence on the core enrichment with a significant correlation to the M0 group. The enrichment plot is presented in Fig. 3 and the heat map in Fig. 4 summarizes all 18 genes including the 5 core enrichment genes.

### Gene set enrichment analysis of PER vs. M0

In addition, to evaluate differences in gene expression levels of patients with peritoneal carcinomatosis (PER) compared to patients without distant metastases (M0), their gene expression results were analyzed. Thus, a GSEA of the NanoString results derived from the use of the nCounter^®^PanCancer Progression Panel was done. However, this analysis did not show a significant downregulation or upregulation of distinct gene sets. For this analysis, the seven above mentioned databases were used.

## Discussion

### HEP vs. M0: reactome toll-like receptor-cascade

Chronic inflammation, as seen in patients suffering from colitis ulcerosa, is associated with an increased risk of CRC. Thus, a chronic inflammatory state, inter alia mediated by an up- or downregulation of TLRs, is closely related to the development of CRC (Pretzsch et al. 2019; Fukata et al. 2007). However, only little is known about the influence of immunological processes on the development of distant metastases (Rumba et al. 2018). In this respect, the present analysis demonstrated a significant overrepresentation of an immunological gene set consisting of 17 genes. Gene group was enriched in tumors of patients with liver metastases compared to patients without distant spread. One of the core enriched genes of the present analysis was the co-chaperon HSP90B1, which directly influences the functionality of most TLRs (Graustein 2018). Recently, it was demonstrated that TLR2 was highly upregulated in CRC compared to normal mucosa (Paarnio et al. 2019) and TLRs might even prove to be important as diagnostic markers. Furthermore, TLRs correlate with high inflammation and poor prognosis in CRC patients (Paarnio et al. 2017). The findings of the present analysis may indicate a convincing relationship between inflammatory processes mediated by TLR-cascades and the metastatic pattern of CRC thus explaining the poor prognosis. Recently, it could be shown in a murine model that the activation of TLR4 resulted in a higher adhesion of tumor cells to the extracellular matrix and increased invasion modulated by NF-κB and ß-integrins, which in turn could imply a higher potential for distant metastasis formation (Wang et al. 2003). In addition, via activation of myeloid differentiation primary response protein 88 (MyD88) initiated by NF-κB and mitogen-activated protein kinases (MAPK) the activation of the TLR cascade led to increased levels of interleukin-6 (IL-6) and 8 (Waugh and Wilson 2008; Kawai et al. 2004). In this respect, an increase in IL-6 was associated with larger tumor size and liver metastases (Galizia et al. 2002). Likewise, lung cancer cells activate macrophages via TLR2/6 and 9 to produce TNF-**α** and IL-6 resulting in a rapid metastatic progress (Kim et al. 2009). The present findings emphasize a strong correlation of TLRs and the formation of liver metastases. In this respect, the analysis of TLRs might also lead to a multimodal therapeutic approach individually defined for each patient. Furthermore, MAPKAPK3, a core enrichment gene upregulated in HEP, is currently under investigation as an immuno-reactive autoantibody to detect primary CRC in blood samples (Babel et al. 2009; Rosa et al. 2011). The present findings further support this approach and it might even become a screening tool detecting metachronous liver metastases in resected, initially M0 CRC patients.

Interestingly, TMEM100, a core enrichment gene upregulated in the M0 subgroup, is a tumor suppressor gene in non-small cell lung carcinoma as well as in hepatocellular carcinoma. Low TMEM100 levels are associated with poor prognosis and distant metastases in both tumor entities (Han et al. 2017; Ou et al. 2015). The knowledge of the exact role of TMEM100 in CRC is limited and further studies are highly demanded. Nonetheless, the present findings indicate that TMEM100 has a similar function in CRC.

In addition, TLR-9 may play an important role in CRC. In a mouse model, IMO-2055, a TLR 9 agonist, exerted anti-tumor effects by increasing IL-6 and 12 levels with only little inflammatory reaction (Wang et al. 2004; Ojik et al. 2003; Damiano et al. 2006). Moreover, the addition of TLR9 agonists to established chemotherapy regimens had intriguing results. IMO-2055 cooperates with EGF, HER2 and VEGF receptor inhibitors and increases their affinity to the receptors. It can also inhibit the growth of KRAS mutated colorectal and pancreatic cancer in combination with cetuximab (Rosa et al. 2011; Damiano et al. 2006; Damiano et al. 2007; Damiano et al. 2009; Basith et al. 2012). Thus, a clinical study currently investigates the effect of the addition of IMO-2055 to 5-Fluorouracil, folin-acid, irinotecan (FOLFIRI) and cetuximab in CRC patients progressing under chemotherapy (Chan et al. 2015). In this respect, the present findings suggest that the cross-talk within a network of genes might be a potential therapeutic target.

### HEP vs. M0: immunologic signature: GSE6875_TCONV_VS_FOXP3_KO_TREG_DN

TILs and their influence on patient survival have been controversially debated over recent years (Shang et al. 2015; Galon et al. 2006; Bösch et al. 2019b). Cytotoxic T-cells have a positive influence on patient survival and serve as a positive prognostic marker (Chiba et al. 2004; Pages et al. 2005). Regulatory T-cells (Tregs), on the contrary, are associated with poor prognosis in most solid tumors (Zou 2006; Needham et al. 2006). However, Tregs are correlated with a better overall survival in patients with CRC (Salama et al. 2009). FOXP3 is the most important transcription factor of Tregs regulating their integrity and function (Marzano et al. 2009). The GSEA of this study demonstrated a significant overrepresentation of genes included in the pathway GSE6875_TCONV_VS_FOXP3_KO_TREG_DN which leads to higher levels of FOXP3 + Tregs when comparing M0 to HEP. Consequently, this suggests that immunosurveillance mediated by FOXP3 might play an important role in preventing distant metastases of CRC. This is further supported by Vlad et al. who demonstrated that stage I or II CRC had significantly more FOXP3 + Tregs than stage III or IV tumors suggesting an anti-tumor effect (Salama et al. 2009; Vlad et al. 2015). Nonetheless, Kim et al. showed that CRC cells are capable to produce FOXP3 + TILs. While the authors confirmed the positive prognostic impact of FOXP3 + TILs on survival, they also underlined that CRC cells can express FOXP3 on their surface, thus avoiding destruction by the host immune system (Kim 2013). In this respect, further studies on the effect of FOXP3 + TILs and CRC are highly demanded. Their influence on survival and their prognostic impact has to be elucidated.

### PER vs. M0

As mentioned above, no gene set contributed to any of the pathways tested in this enrichment analysis. This limitation is most likely due to the limited number of genes resulting from the NanoString analysis. As the GSEA approach rather evaluates the workflow of a whole set of genes and its role in any pathway of interest, a certain number of input genes is required to be able to gain significant insights. Since the majority of pathways analyzed consist of a rather large number of genes, these criteria were probably not met. Nonetheless, this lack of significance further supports the hypothetical approach of a biological difference in tumors leading either to liver or peritoneal metastases.

Although the present findings indicate a relationship between immune related gene sets and organotropism in CRC patients, there are limitations as well. Firstly, this study is based on a correlative level applying the nCounter^®^PanCancer Progression Panel by NanoString-Technologies. The panel allows a comprehensive analysis of almost 800 metastasis-related genes, but not a functional relationship. Nonetheless, this demanding assay and the large-scale GSEA helps to elucidate a correlation between gene sets, metastasis and organotropism. Secondly, the study includes 18 patients, which represents a small collective at a first glance. However, the large-scale GSEA should identify gene sets associated to metastasis. Moreover, the present study was designed as a basis for investigating organotropism in colorectal cancer in future studies. In this regard, in a first step the analysis of a clear and well-characterized cohort facilitates the identification of distinct differences more easily. Therefore, based on the present intriguing findings further studies are urgently needed to analyze the functional relevance of the identified gene sets. The present findings should thereby be the basis for further research.

In conclusion, the present study shows that the metastatic route to the liver is partially driven by immunologic reactions. It seems that TLRs play an important role, but the exact role of TLRs needs to be elucidated. Since distant metastases are the main driver of CRC-related death, TLR-associated pathways represent a promising approach for diagnosis and treatment. Moreover, it seems that the upregulation of Tregs mediated by FOXP3 at least in part hindered primary CRC from forming distant metastases. This probably illustrates an adjuvant treatment possibility for non-metastasized patients.

## Abbreviations

CRC: Colorectal cancer
EMT: Epithelial mesenchymal transition
FAP: Familiarly adenomatous polyposis
FFPE: Formalin-fixed paraffin-ebedded tissue
FOLFIRI: 5-Fluorouracil, folin-acid, irinotecan
FOXP3: Fork-head/winged-helix transcription factor 3
GO: Gene Ontology
GSEA: Gene set enrichment analysis
HNPCC: Hereditary non-polyposis colon cancer
IL: Interleukin
KEGG: Kyoto Encyclopedia of Genes and Genomes
MAPK: Mitogen-activated protein kinase
NF-κB: Nuclear factor kappa-light-chain-*enhancer of activated B-cells*
NES: Normalized enrichment score
TILs: Tumor infiltrating lymphocytes
TLR: Toll-like receptors
TNF-α: *Tumor Necrosis Factor-α*
Tregs: CD4/25 + regulatory T-cells
VEGF: Vascular-endothelial growth-factor
